# Relationship between the preoperative AST/ALT ratio and postoperative delirium and 3-year mortality in patients undergoing total knee or hip arthroplasty: a prospective cohort study

**DOI:** 10.1097/JS9.0000000000003914

**Published:** 2025-11-11

**Authors:** Jian Kong, Shanling Xu, Yuanlong Wang, Shuhui Hua, Hongyan Gong, Rui Dong, Jiahan Wang, Yanan Lin, Chuan Li, Yanlin Bi, Bin Wang, Xu Lin

**Affiliations:** a School of Anesthesiology, Shandong Second Medical University, Weifang, China; b The Second School of Clinical Medicine, Binzhou Medical University, Yantai, China; c Department of Anesthesiology, Qingdao Municipal Hospital, Qingdao, Shandong, China

**Keywords:** Alzheimer’s disease biomarkers, AST/ALT ratio, cerebrospinal fluid, liver enzymes, postoperative delirium

## Abstract

**Objective:**

To investigate the association between the preoperative serum aspartate aminotransferase to alanine aminotransferase (AST/ALT) ratio and postoperative delirium (POD) and 3-year mortality in POD patients.

**Methods:**

A total of 538 clinical participants were enrolled from the Perioperative Neurocognitive Disorder and Biomarkers Lifestyle (PNDABLE) study. In this study, patients were first categorized into a POD group and a non-POD group, and the demographic characteristics of the two groups were compared. Preoperative serum AST and ALT levels were measured to calculate the AST/ALT ratio. Cerebrospinal fluid (CSF) Alzheimer’s disease (AD)-related biomarkers were analyzed. Logistic regression and sensitivity analyses were performed to identify protective and risk factors for POD. Mediation effect models were applied to evaluate the potential mediating roles of CSF biomarkers. Receiver operating characteristic curves and decision curve analysis were used to validate predictive performance. Restricted cubic spline (RCS) regression was employed to explore the dose–response relationship between AST/ALT levels and POD risk. Finally, patients with POD were followed up for 3 years, and Kaplan–Meier (K–M) survival analysis was used to compare the mortality rates of the AST/ALT ratio in patients with POD.

**Results:**

The incidence of POD was 17.53%. Logistic regression revealed that an elevated preoperative AST/ALT ratio was an independent risk factor for POD [odds ratio (OR) = 4.305, 95% confidence interval (CI) 2.404–7.706, *P* < 0.001]. Reduced CSF amyloid-beta 42 (Aβ42) levels (OR = 0.992, 95% CI 0.990–0.994, *P* < 0.001) were inversely associated with POD risk, whereas elevated total tau (OR = 1.016, 95% CI 1.012–1.019, *P* < 0.001) and phosphorylated tau (P-tau) (OR = 1.097, 95% CI 1.069–1.127, *P* < 0.001) were identified as risk factors. Sensitivity and *post hoc* analyses supported these findings. Mediation analysis demonstrated that the effect of AST/ALT on POD was partially mediated by CSF Aβ42 (12.9%) and the Aβ42/P-tau ratio (14.8%). The predictive model combining AST/ALT with CSF biomarkers achieved optimal performance (area under the curve = 0.92). Follow-up of 88 patients showed that K–M survival analysis revealed that although mortality rates were higher in patients with POD, there were no significant differences in 3-year survival rates between the high AST/ALT ratio group and the low AST/ALT ratio group.

**Conclusion:**

Preoperative elevation of the serum AST/ALT ratio is a potential risk factor for POD, and its effect may be partially mediated by AD-related CSF biomarkers. However, an increase in the AST/ALT ratio did not have a significant effect on the three-year survival rate of POD patients.

Postoperative delirium (POD), a critical acute complication and subtype of postoperative neurocognitive disorders^[1]^, typically manifests within 7 days postoperatively or prior to discharge^[2,3]^. It is characterized by fluctuating symptoms, including inattention, disorganized thinking, and cognitive dysfunction^[4,5,6]^. As one of the most prevalent postoperative complications – particularly among elderly patients – POD significantly compromises long-term prognosis, quality of life, and healthcare costs^[7]^. Elucidating its pathogenesis and risk factors is therefore essential for reducing POD incidence.

The liver, a central hub for systemic metabolism, facilitates amyloid-beta 42 (Aβ42) clearance via metabolic enzyme activity, bile secretion, and hepatocyte surface receptor-mediated pathways^[8]^. Hepatic function critically determines protein clearance efficiency, with the aspartate aminotransferase to alanine aminotransferase (AST/ALT) ratio serving as a key indicator of hepatic integrity^[9,10]^. Elevated AST/ALT ratios reflect hepatic dysfunction, which disrupts physiological homeostasis, energy metabolism, and macromolecule synthesis/clearance^[11]^. Emerging evidence positions liver function indices (e.g., AST/ALT) as potential biomarkers for cognitive impairment^[11,12]^. An elevated AST/ALT ratio may also indicate enhanced oxidative stress, which may impair neuronal function and hepatocyte clearance through degradation pathways and biliary excretory mechanisms^[12]^.

While the precise etiology of POD remains debated, prior studies implicate blood–brain barrier (BBB) disruption and neuroinflammatory responses in its development^[13,14]^. Notably, reduced cerebrospinal fluid (CSF) Aβ42 levels and elevated total tau (T-tau) concentrations have been mechanistically linked to delirium states, suggesting a neurodegenerative component in POD pathology^[15,16]^.

This investigation aims to evaluate the association between preoperative AST/ALT ratios and POD incidence in patients undergoing total knee/hip arthroplasty, and 3-year survival of POD patients was analyzed, providing insights for early prevention strategies targeting perioperative cognitive decline.

This prospective cohort study has been reported in line with the STROCSS 2025 guidelines.

## Material and Methods

### PNDABLE database

All clinical participants in this study were enrolled from the Perioperative Neurocognitive Disorder and Biomarker Lifestyle (PNDABLE) study, a prospective, large-scale cohort study investigating risk factors and biomarkers in Northern Han Chinese populations. The inclusion criteria stipulated participants aged between 40 and 90 years. This research protocol, adhering to the ethical principles outlined in the Declaration of Helsinki, has been approved by the Ethics Committee and registered with the Chinese Clinical Trial Registry.

All enrolled clinical participants provided comprehensive informed consent after being thoroughly apprised of study procedures, including biological sample collection (peripheral blood and CSF). Clinical participants retain the unconditional right to withdraw from the study at any time without penalty or compromise to their clinical care.

### Participants

A total of 538 consecutive clinical participants scheduled for elective total knee or hip arthroplasty under combined spinal–epidural anesthesia at Qingdao Municipal Hospital between February 2021 and February 2022 were enrolled. All participants provided written informed consent prior to surgery and underwent comprehensive clinical and neuropsychological evaluations 1 day preoperatively. Demographic data were collected through structured questionnaires, electronic medical record systems, and laboratory information management systems. Inclusion criteria are the following: (1) age ≥40 years, (2) Ethnic Han Chinese from northern China, (3) American Society of Anesthesiologists physical status classifications I–II, (4) Mini-Mental State Examination (MMSE) score >23 with intact verbal communication, and (5) Educational attainment sufficient to complete preoperative cognitive assessments. Exclusion criteria are the following: (1) central nervous system disorders, including intracranial infections, traumatic brain injury, epilepsy, multiple sclerosis, or other major neurological conditions; (2) major psychiatric comorbidities, including depression, delirium, subjective cognitive decline, mild cognitive impairment, or related disorders; (3) severe systemic diseases that may alter CSF biomarker levels (e.g., malignancies); (4) family history of genetic disorders; (5) chronic use of psychotropic agents, corticosteroids, or hormone therapies; (6) recent major surgical history (within 3 months); (7) significant visual/hearing impairments compromising cognitive evaluation; (8) preoperative coagulation abnormalities (INR >1.5 or platelet count <100 × 10^9^/L); and (9) the Child–Pugh classification (key parameters include serum bilirubin, serum albumin (ALB), prothrombin time, presence of ascites, and hepatic encephalopathy) was categorized as Class C.

**HIGHLIGHTS**

- The aspartate aminotransferase to alanine aminotransferase (AST/ALT) ratio is independently associated with an increased risk of postoperative delirium (POD).
- Cerebrospinal fluid amyloid-beta 42 (Aβ42) and Aβ42/P-tau mediate the association between AST/ALT and POD.
- The increase in the AST/ALT, the risk of POD increased linearly, and the results were still significant after classification by gender and correction of confounding factors.

### Outcome measures

Primary outcome was whether the increase in the AST/ALT index of liver function was a protective or risk factor for POD. Secondary outcomes were whether the effect of AST/ALT on POD was mediated by CSF-related biomarkers and whether the 3-year survival rate of patients diagnosed with POD is related to AST/ALT, a preoperative liver function index.

### Neuropsychological testing

POD was diagnosed using the Confusion Assessment Method (CAM), and its severity was quantified with the Memorial Delirium Assessment Scale (MDAS). Preoperative cognitive screening was performed using the MMSE, with patients scoring >23 points and demonstrating intact verbal communication eligible for inclusion. All evaluations were conducted by a multidisciplinary team comprising board-certified anesthesiologists and neurologists. Preoperative and postoperative assessments were performed by two independent physicians to minimize observer bias. The evaluation time of this study was 1 day before surgery and the 1st, 3rd, 5th, and 7th days after surgery, or before discharge. The CAM and MDAS instruments were selected for their established reliability and broad applicability in perioperative settings.

### Anesthesia and surgery

Clinical participants undergoing total knee/hip arthroplasty adhered to standard fasting protocols, with no premedication administered. Standard monitoring was initiated upon operating room arrival, combined spinal–epidural anesthesia was performed, supplemental oxygen was administered via face mask, and vasoactive agents were titrated to maintain hemodynamic stability. Patients were transferred to the post-anesthesia care unit for 30-min observation prior to ward discharge, contingent upon stable recovery parameters (Supplemental Digital Content S1, available at: http://links.lww.com/JS9/F528).

### CSF core biomarker collection and measurements

Fasting venous blood samples (3 mL) were collected preoperatively. Serum AST and ALT levels, along with AST/ALT ratios, were quantified using an enzymatic coupled assay on an automated biochemical analyzer (AU5800, Beckman Coulter, USA). CSF samples obtained during dural puncture were processed within 2 hours post-collection: all samples were centrifuged at 2000 × g for 10 min at 4°C, and then supernatant aliquoted into non-enzymatic tubes (Eppendorf) and immediately stored at −80°C until analysis. CSF Aβ42, T-tau, and phosphorylated tau (P-tau) concentrations were measured using enzyme-linked immunosorbent assay kits (Thermo Scientific Multiskan MK3 microplate reader). Aβ42/P-tau and Aβ42/T-tau ratios were subsequently calculated. All assays were performed by certified laboratory technicians using standardized protocols, and blinded allocation of samples ensured analytical objectivity. All antibodies and culture plates originated from a single manufacturing lot to eliminate inter-batch variability.

### Follow-up

Survival duration was defined as the period from the surgical procedure date to either the death event or the final follow-up documentation. Researchers monitored clinical participants with POD throughout a 3-year observational phase following their operation.

### Sample size estimation

According to previous literature reports, the incidence of POD was 17.5%, and the study design anticipated a 15% loss to follow-up rate. Based on the “events per variable” (EPV) sample size calculation method for logistic regression models^[17]^, with the EPV parameter set to 10, the formula-derived required sample size was 538 cases (8 × 10 ÷ 0.175 ÷ 0.85 = 538)^[18]^.

### Statistical analysis

SPSS 25.0 (IBM SPSS Inc., Chicago, IL, USA), R software version 4.3.1 (R Foundation for Statistical Computing, Vienna, Austria), and Stata MP16.0 (Solvu Soft Corporation, Inc., Chicago, IL, USA) were used for data analysis. The standard with significance was *P* < 0.05.

In demographic analyses, the Kolmogorov–Smirnov test was employed to assess the normality of continuous variables. Normally distributed data were expressed as mean ± standard deviation (SD) and compared using the two-independent-samples *t*-test. Non-normally distributed data were reported as median and interquartile range (*M*, Q25–Q75) and analyzed via the Mann–Whitney *U* test. Categorical variables were evaluated using chi-square/Fisher’s exact tests. AST/ALT was investigated as a composite factor to explore its association with POD.

Univariate binary logistic regression analyses were performed to identify risk and protective factors for POD by separately evaluating AST/ALT, Aβ42, T-tau, P-tau, Aβ42/T-tau, and Aβ42/P-tau ratios. Subsequently, to mitigate confounding effects, we conducted multiple sensitivity analyses in this study by constructing three adjusted models: (1) Model 2 adjusted for age, sex, years of education, and MMSE scores; (2) Model 3 additionally adjusted for hypertension, diabetes, smoking, and alcohol consumption history; and (3) Model 4 further restricted to participants the body mass index (BMI) limit ≤28 kg/m^2^ based on Model 3. Given that multiple comparisons may increase the risk of type I error, the Bonferroni method was used to correct the *P* values with a correction threshold of α/*n* (*n* is the number of independent tests).

Mediation analysis was performed using Stata MP 18.0 to investigate whether the association between the AST/ALT ratio and POD was mediated by CSF-related biomarkers (Aβ42, T-tau, P-tau, Aβ42/T-tau, and Aβ42/P-tau). A statistically significant mediation effect was established if the following criteria were concurrently met: (1) the association between AST/ALT and POD was attenuated after CSF biomarkers were included in the mo (2) CSF biomarkers were significantly associated with POD; (3) AST/ALT was significantly associated with POD; and (4) AST/ALT was significantly associated with CSF biomarkers.

Taking it a step further, the predictive value of the AST/ALT ratio and CSF-related biomarkers was evaluated using R software version 4.3.1. Receiver operating characteristic (ROC) curves were plotted to describe diagnostic performance, with the area under the curve (AUC) calculated to report discriminatory capacity. The DeLong test was used to compare the AUC. Decision curve analysis (DCA) was performed to assess the clinical utility of AST/ALT and CSF biomarker models in predicting POD.

Restricted cubic splines (RCSs) were plotted using R software 4.3.1 to evaluate the dose–response association between the AST/ALT ratio and POD risk. Subgroup analyses stratified by gender were conducted to examine heterogeneity in this association.

*Post hoc* analysis was performed, excluding patients with age ≤70, years of education ≤9, and BMI ≥28, to explore whether the study results were applicable to individuals with older, more educated, and non-obese individuals.

Finally, Kaplan–Meier survival analysis was performed to assess the effect of different subgroups on the 3-year mortality in POD patients, with the Log-rank test used to evaluate survival risk differences between groups.

### Statement

The work has been reported in line with the STROCSS 2025 criteria^[19]^.

## Result

### Participant characteristics

**Figure 1.**
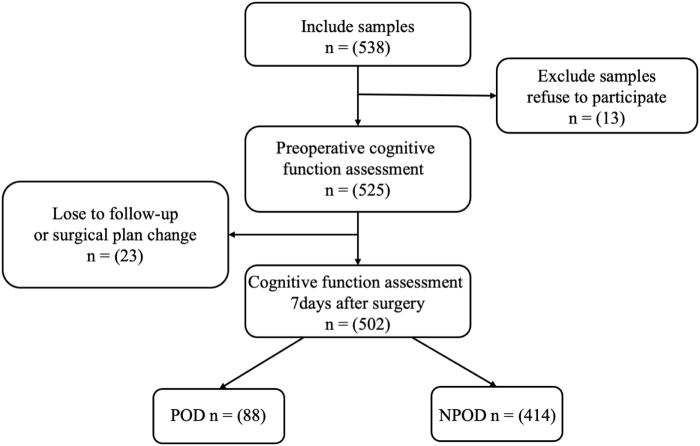
The flow diagram showed the selection of eligible patients and the enrollment process.

A total of 538 eligible clinical participants were screened, with 502 ultimately enrolled in the PNDABLE study (Fig. 1). Among them, 88 participants developed POD within 7 days postoperatively or prior to discharge, yielding a POD incidence rate of 17.53%.

Comparative analysis between the POD and non-POD (NPOD) groups revealed no significant differences in sex, BMI, MMSE scores, history of hypertension/diabetes, smoking/alcohol consumption, anesthesia duration, operative time, intraoperative fluid administration, or blood loss. Statistically significant differences (*P* < 0.001) were observed in CSF biomarkers and ratios (Aβ42, T-tau, P-tau, Aβ42/T-tau, and Aβ42/P-tau), age, years of education, and AST/ALT ratio between the two groups (Table 1). Analysis of baseline data of total knee arthroplasty and total hip arthroplasty showed that the incidence of age (*P* = 0.031), gender, surgery time, anesthesia, infusion volume, bleeding volume, and POD differed significantly between different surgeries (*P* <0.001) (Table 2).

**Table 1:** The comparison of two groups in the general condition and the perioperative condition.

	POD	NPOD	*P*
Number	88	414	
Age, *M* (IQR), years	73.50 (66.25–77.00)	63.00 (57.00–67.00)	**<0.001**
Male (%)	31 (35.20%)	160 (38.60%)	0.629
Education, *M* (IQR), years	9.00 (5.00–9.00)	12.00 (9.00–12.00)	**<0.001**
BMI, *M* (IQR), kg/m^2^	26.00 (24.00–28.00)	25.00 (23.00–28.00)	0.159
MMSE score, *M* (IQR)	29.00 (27.00–30.00)	28.00 (27.00–30)	0.542
Cigarette consumption, yes (%)	17 (19.30%)	116 (28.00%)	0.110
Alcohol habit, yes (%)	26 (29.50%)	145 (35.00%)	0.386
Hypertension, yes (%)	39 (44.30%)	143 (34.50%)	0.088
Diabetes, yes (%)	18 (20.50%)	61 (14.70%)	0.197
Duration of surgery, *M* (IQR), h	1.42 (1.00–2.00)	1.58 (0.83–2.08)	0.337
Duration of anesthesia, *M* (IQR), h	2.29 (1.75–2.83)	2.42 (1.67–3.08)	0.524
Infusion volume, *M* (IQR), ml	1100.00 (600.00–1100.00)	1100.00 (600.00–1100.00)	0.225
Bleeding volume, *M* (IQR), ml	20.00 (5.00–125.00)	20.00 (5.00–150.00)	0.925
AST/ALT, *M* (IQR)	1.17 (1.01–1.46)	1.07 (0.88–1.30)	**<0.001**
CSF biomarkers, *M* (IQR), pg/ml			
CSF Aβ42	292.40 (229.92–375.72)	397.80 (298.06–523.22)	**<0.001**
CSF T-tau	320.56 (233.28–271.49)	180.25 (143.06–227.68)	**<0.001**
CSF P-tau	49.88 (40.82–55.82)	39.44 (32.66–46.64)	**<0.001**
Ratios of biomarkers, *M* (IQR)			
CSF Aβ42/T-tau	0.96 (0.70–1.50)	2.18 (1.57–3.03)	**<0.001**
CSF Aβ42/P-tau	6.17 (4.31–7.90)	10.31 (7.46–13.73)	**<0.001**

BMI, body mass index; CSF, cerebrospinal fluid; IQR, interquartile range; *M*, median; MMSE, Mini-Mental State Examination; NPOD, no postoperative delirium; POD, postoperative delirium.

Categorical variables are reported as numbers and percentages; continuous variables are reported as means ± SDs, whereas non-normal data are expressed as *M* (Q25–Q75).

The bold values are all *P* ≤ 0.050, statistically significant parameters.

**Table 2:** Comparison of TKA and THA in general with perioperative conditions.

	TKA	THA	*P*
Number	411	91	
POD, yes (%)	65 (15.80%)	23 (25.30%)	**0.046**
Age, *M* (IQR), years	64.00 (58.00–68.00)	60.00 (60.00–70.00)	**0.031**
Male (%)	139 (33.80%)	52 (57.10%)	**<0.001**
Education, *M* (IQR), years	9.00 (9.00–12.00)	9.00 (9.00–12.00)	0.722
BMI, *M* (IQR), kg/m^2^	25.00 (23.00–28.00)	26.00 (24.00–28.00)	0.363
MMSE score, *M* (IQR)	28.00 (27.00–30.00)	28.00 (27.00–30.00)	0.466
Cigarette consumption, yes (%)	113 (27.50%)	20 (22.00%)	0.297
Alcohol habit, yes (%)	138 (33.60%)	33 (36.30%)	0.627
Hypertension, yes (%)	148 (36.00%)	34 (37.40%)	0.811
Diabetes, yes (%)	61 (14.80%)	18 (19.80%)	0.265
Duration of surgery, *M* (IQR), h	2.17 (1.58–2.92)	2.00 (1.67–2.42)	**<0.001**
Duration of anesthesia, *M* (IQR), h	1.42 (0.75–2.00)	3.08 (2.58–3.50)	**<0.001**
Infusion volume, *M* (IQR), ml	1000.00 (600.00–1100.00)	1100.00 (1100.00–1600.00)	**<0.001**
Bleeding volume, *M* (IQR), ml	10.00 (5.00–50.00)	300.00 (200.00–300.00)	**<0.001**
AST/ALT, *M* (IQR)	1.07 (0.89–1.27)	1.26 (0.98–1.47)	**<0.001**
CSF biomarkers, *M* (IQR), pg/ml			
CSF Aβ42	384.40 (285.82–504.77)	354.16 (266.70–449.10)	0.077
CSF T-tau	198.50 (149.40–254.87)	190.92 (140.10–253.41)	0.450
CSF P-tau	41.09 (33.71–48.84)	40.14 (33.80–50.71)	0.832
Ratios of biomarkers, *M* (IQR)			
CSF Aβ42/T-tau	1.97 (1.37–2.82)	1.94 (1.29–2.81)	0.603
CSF Aβ42/P-tau	9.42 (6.87–13.03)	8.50 (6.17–13.18)	0.256

BMI, body mass index; CSF, cerebrospinal fluid; IQR, interquartile range; *M*, median; MMSE, Mini-Mental State Examination; POD, postoperative delirium; THA, total hip arthroplasty; TKA, total knee arthroplasty.

Categorical variables are reported as numbers and percentages; continuous variables are reported as means ± SDs, whereas non-normal data are expressed as *M* (Q25–Q75).

The bold values are all *P* ≤ 0.050, statistically significant parameters.

### Protective and risk factors on POD

Univariate logistic regression analysis demonstrated that the AST/ALT ratio [odds ratio (OR) = 4.305, 95% confidence interval (CI) 2.404–7.706, *P* < 0.001], CSF T-tau (OR = 1.016, 95% CI 1.012–1.019, *P* < 0.001), and P-tau (OR = 1.097, 95% CI 1.069–1.127, *P* < 0.001) were risk factors for POD, while Aβ42 (OR = 0.992, 95% CI 0.990–0.994, *P* < 0.001), Aβ42/T-tau (OR = 0.123, 95% CI 0.075–0.202, *P* < 0.001), and Aβ42/P-tau (OR = 0.668, 95% CI 0.601–0.743, *P* < 0.001) served as protective factors (Table 3, Model 1). Furthermore, sensitivity analyses were performed to validate the robustness of the findings. Based on binary logistic regression analysis adjusted for age, sex, years of education, MMSE scores, hypertension, diabetes, and histories of smoking and alcohol consumption (Table 3, Model 3), elevated AST/ALT ratio (OR = 8.933, 95% CI 4.173–19.397, *P* < 0.001) remained an independent risk factor for POD. Finally, subgroup analysis restricted to baseline individuals with a BMI limit of ≤ 28 kg/m^2^ confirmed that an elevated AST/ALT ratio (OR = 6.495, 95% CI 2.544–16.584, *P* < 0.001) persisted as a significant risk factor for POD (Table 3, Model 4).

**Table 3:** Logistic regression analysis of risk factors for POD.

	Model 1		Model 2		Model 3		Model 4	
	OR (95% CI)	*P*	OR (95% CI)	*P*	OR (95% CI)	*P*	OR (95% CI)	*P*
AST/ALT	4.305 (2.404–7.706)	**<0.001**	9.040 (4.256–19.199)	**<0.001**	8.933 (4.173–19.397)	**<0.001**	6.495 (2.544–16.584)	**<0.001**
CSF biomarkers, median (IQR), pg/ml								
Aβ42	0.992 (0.990–0.994)	**<0.001**	0.993 (0.990–0.996)	**<0.001**	0.993 (0.990–0.996)	**<0.001**	0.991 (0.987–0.995)	**<0.001**
T-tau	1.016 (1.012–1.019)	**<0.001**	1.012 (1.009–1.016)	**<0.001**	1.012 (1.009–1.016)	**<0.001**	1.014 (1.009–1.018)	**<0.001**
P-tau	1.097 (1.069–1.127)	**<0.001**	1.077 (1.045–1.110)	**<0.001**	1.078 (1.044–1.112)	**<0.001**	1.083 (1.044–1.124)	**<0.001**
Ratios of biomarkers, median (IQR)								
Aβ42/T-tau	0.123 (0.075–0.202)	**<0.001**	0.197 (0.120–0.324)	**<0.001**	0.208 (0.126–0.344)	**<0.001**	0.164 (0.088–0.306)	**<0.001**
Aβ42/P-tau	0.668 (0.601–0.743)	**<0.001**	0.723 (0.647–0.808)	**<0.001**	0.719 (0.641–0.806)	**<0.001**	0.683 (0.592–0.788)	**<0.001**

95% CI, 95% confidence interval; OR, odds ratio.

Model 1: the unadjusted logistic regression.

Model 2: sensitivity analysis was based on more covariates, including age, gender, years of education, and MMSE.

Model 3: on the basis of Model 2, the history of diabetes, hypertension, smoking, and alcohol consumption was added to the sensitivity analysis.

Model 4: on the basis of Model 3, the BMI limit is ≤28 kg/m^2^.

The bold values are all *P* ≤ 0.050, statistically significant parameters.

### Mediation analysis

**Figure 2.**
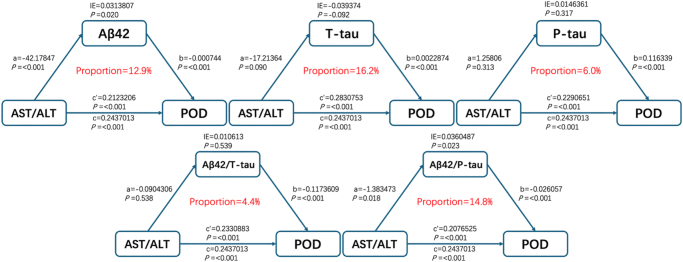
Analysis of the mediating effect of AST/ALT. IE is an indirect effect of the mediating effect; AST/ALT is aspartate aminotransferase/alanine aminotransferase; POD is postoperative delirium; Aβ42 is β amyloid; T-tau is the total tau protein; and p-tau is a phosphorylated tau protein.

The aforementioned findings suggest that the AST/ALT ratio and related CSF biomarkers were risk factors for POD. We subsequently investigated whether AST/ALT contributes to POD by modulating CSF-associated biomarkers (Fig. 2). Mediation analysis revealed that CSF Aβ42 (mediation proportion: 12.9%, *P* = 0.020) and Aβ42/P-tau ratio (mediation proportion: 14.8%, *P* = 0.023) partially mediated the association between AST/ALT and POD.

### Post hoc *analyses*

The PNDABLE cohort was studied after excluding patients with age ≤70, years of education ≤9, and BMI ≥28. The findings were as follows: Model 1 [AST/ALT (OR, 24.945; 95% CI: 7.368–84.454; *P* < 0.001)]; Model 2 [AST/ALT (OR, 21.637; 95% CI: 2.637–117.509; *P* < 0.001)]; Model 3 [AST/ALT (OR, 9.133; 95% CI: 3.293–25.335; *P* < 0.001)], indicating that our results remained stable, suggesting that AST/ALT as a risk factor for POD (Table 4).

**Table 4:** *Post hoc* analyses in the PNDABLE study.

	Model 1		Model 2		Model 3	
	OR (95% CI)	*P*	OR (95% CI)	*P*	OR (95% CI)	*P*
AST/ALT	24.945 (7.368–84.454)	**<0.001**	21.637 (2.637–177.509)	**0.004**	9.133 (3.293–25.335)	**<0.001**
CSF biomarkers,median (IQR), pg/ml						
Aβ42	0.986 (0.971–1.001)	**0.070**	0.989 (0.969–1.009)	0.267	0.995 (0.982–1.008)	0.469
T-tau	1.032 (1.019–1.046)	**<0.001**	1.025 (1.011–1.039)	**<0.001**	1.027 (1.017–1.036)	**<0.001**
P-tau	1.033 (0.902–1.184)	0.636	1.093 (0.907–1.318)	0.305	0.984 (0.874–1.108)	0.793
Ratios of biomarkers, median (IQR)						
Aβ42/T-tau	4.614 (1.058–20.121)	**0.042**	6.020 (1.274–28.457)	**0.024**	3.511 (1.159–10.636)	0.026
Aβ42/P-tau	0.862 (0.463–1.604)	0.639	0.856 (0.336–2.180)	0.744	0.668 (0.376–1.188)	0.169

OR, odds ratio; CI, confidence interval.

Model 1: exclude age ≤ 70.

Model 2: exclude years of education ≤ 9

Model 3: exclude BMI ≥ 28.

The bold values are all *P* ≤ 0.050, statistically significant parameters. Models 1–3 refer to the model of *post hoc* analyses.

### ROC curve analysis

**Figure 3.**
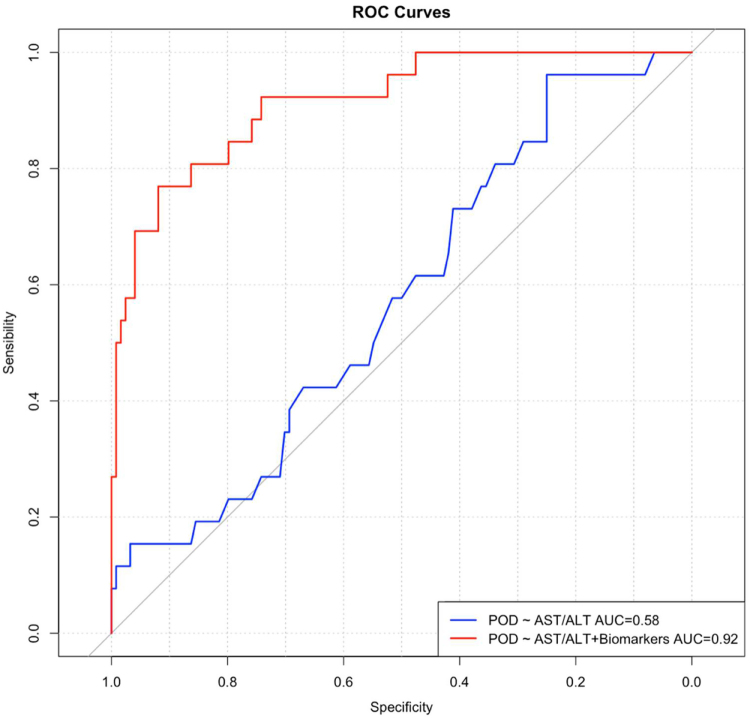
ROC curves of AST/ALT combined with CSF biomarkers for predicting POD.

**Figure 4.**
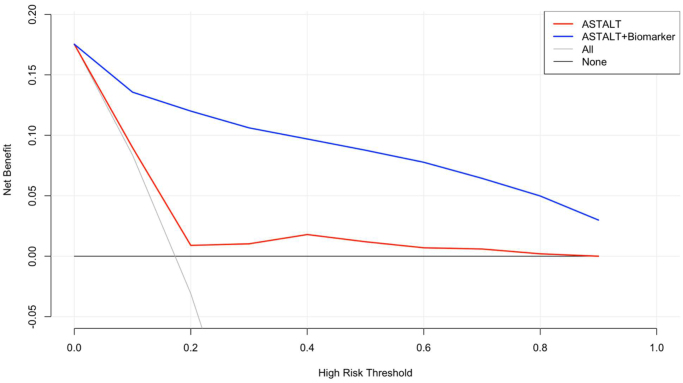
DCA curves of AST/ALT combined with CSF biomarkers in predicting POD.

The ROC curve demonstrated that the combined model integrating AST/ALT ratios with CSF biomarkers (Aβ42, T-tau, P-tau) exhibited superior discriminatory performance in predicting POD (AUC = 0.92) compared to the AST/ALT ratio alone (AUC = 0.58) (Fig. 3). The DCA revealed that the AST/ALT-based POD prediction model showed clinical utility only within the low threshold probability range (0.0–0.2) based on net benefit area. The Delong test was used to compare the AUC, and it was found that the AUC predicted by ASL/ALT combined with CSF biomarkers was higher than that predicted by ASL/ALT alone, and the difference was statistically significant (*z* = 9.873, *P* < 0.001). The combined model (AST/ALT + CSF biomarkers) maintained robust performance across both low and high threshold probability ranges, achieving significantly greater net benefit than the AST/ALT-only model. This enhanced predictive accuracy suggests stronger potential for guiding clinical decision-making (Fig. 4).

### RCS analysis

**Figure 5.**
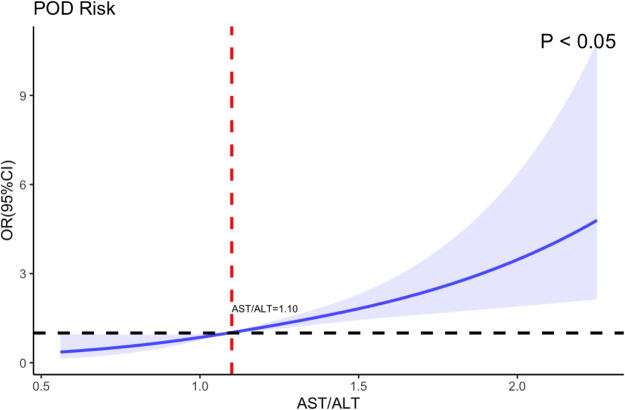
Restricted cubic spline curve of the relationship between AST/ALT and POD without adjustment.

**Figure 6.**
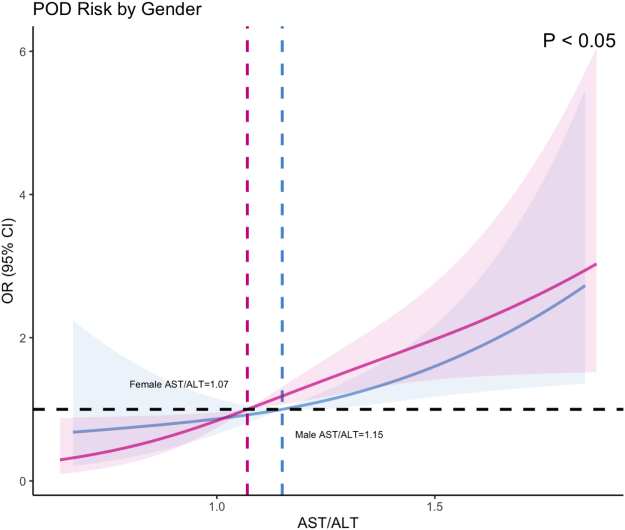
Restricted cubic spline curve of the relationship between AST/ALT and POD stratified by gender.

**Figure 7.**
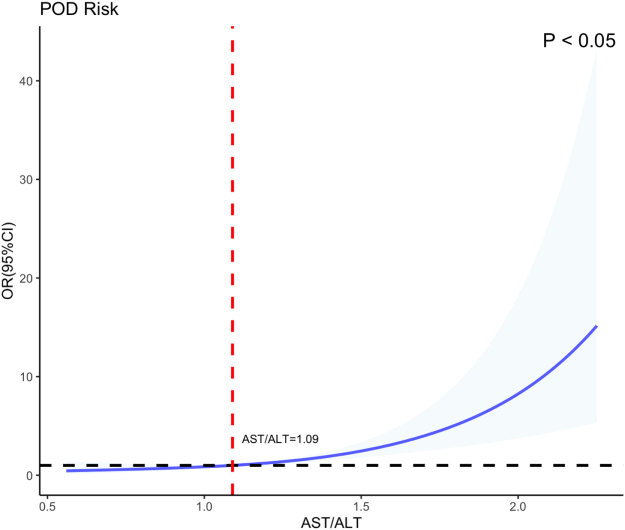
The restricted cubic spline curve for the relationship between the corrected AST/ALT and POD.

RCS curves were employed to evaluate the dose–response relationship between AST/ALT levels and POD. The unadjusted RCS analysis revealed a positive association between AST/ALT levels and POD risk (*P* < 0.05), demonstrating an overall linear dose–response relationship. Specifically, POD risk remained relatively low when the AST/ALT <1.10, but significantly increased when the ratio exceeded this threshold (Fig. 5). Gender-stratified RCS curves showed comparable associations between AST/ALT levels and POD probability (*P* < 0.05), confirming the robustness of the observed relationship across subgroups (Fig. 6). After adjusting for age, education level, gender, MMSE score, hypertension, diabetes mellitus, smoking history, alcohol consumption, and BMI, the RCS curves maintained a linear correlation between AST/ALT levels and POD risk. A critical threshold was identified at an AST/ALT ratio of 1.09, beyond which POD risk escalated significantly (*P* < 0.05) (Fig. 7). The baseline data of the two groups were compared (Table 5).

**Table 5:** Baseline characteristics of patients above vs below a cut-off of AST/ALT.

	AST/ALT < 1.09	AST/ALT ≥ 1.09	*P*
Number	246	256	
POD, yes (%)	33 (13.40%)	55 (21.50%)	**0.017**
Age, *M* (IQR), years	63.00 (57.00–68.00)	65.00 (60.00–70.00)	**0.001**
Male (%)	84 (34.10%)	107 (41.80%)	0.078
Education, *M* (IQR), years	9.00 (9.00–12.00)	9.00 (9.00–12.00)	0.886
BMI, *M* (IQR), kg/m^2^	23.00 (25.00–27.25)	25.00 (23.00–28.00)	0.539
MMSE score, *M* (IQR)	28.00 (27.00–30.00)	28.00 (27.00–30.00)	0.773
Alcohol habit, yes (%)	93 (37.80%)	78 (30.50%)	0.083
Hypertension, yes (%)	80 (32.50%)	102 (39.80%)	0.088
Diabetes, yes (%)	46 (18.70%)	33 (12.90%)	0.074
Duration of surgery, *M* (IQR), h	1.58 (0.92–2.17)	1.50 (0.83–2.00)	0.264
Duration of anesthesia, *M* (IQR), h	2.42 (1.75–3.17)	2.42 (1.67–3.00)	0.285
Infusion volume, *M* (IQR), ml	1100.00 (600.75–1200.00)	1100.00 (600.00–1100.00)	**0.042**
Bleeding volume, *M* (IQR), ml	20.00 (10.00–100.00)	20.00 (5.00–200.00)	0.178
CSF biomarkers, *M* (IQR), pg/ml			
CSF Aβ42	381.05 (285.42–523.22)	378.12 (282.23–471.29)	0.264
CSF T-tau	201.43 (152.02–261.14)	188.54 (142.81–246.10)	0.164
CSF P-tau	39.82 (34.07–48.34)	41.93 (33.56–49.83)	0.433
Ratios of biomarkers, *M* (IQR)			
CSF Aβ42/T-tau	1.95 (1.36–2.80)	1.98 (1.37–2.85)	0.838
CSF Aβ42/P-tau	9.72 (6.95–13.18)	9.00 (6.48–12.93)	0.164

POD, postoperative delirium; BMI, body mass index; MMSE, Mini-Mental State Examination; *M*, median; IQR, interquartile range; CSF, cerebrospinal fluid.

Categorical variables are reported as numbers and percentages; continuous variables are reported as means ± SDs, whereas non-normal data are expressed as *M* (Q25–Q75).

The bold values are all *P* ≤ 0.050, statistically significant parameters.

### Analysis of 3-year survival rates

The 3-year longitudinal follow-up of 88 patients diagnosed with POD demonstrated a 94.32% overall survival rate, with five mortalities recorded and no instances of participant attrition during the study period.

**Figure 8.**
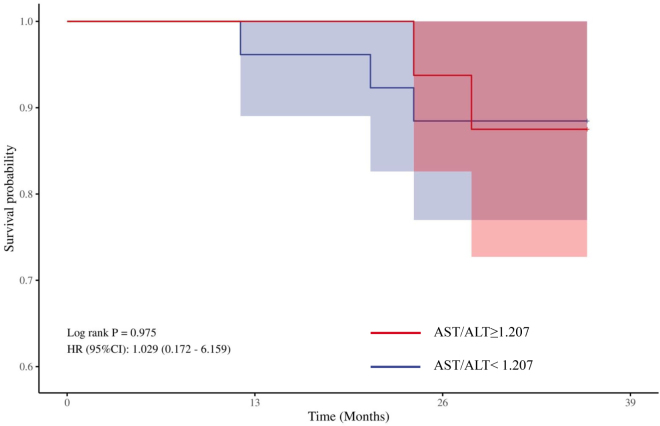
Kaplan–Meier curve: analysis of the 3-year survival status of patients with POD.

In participants diagnosed with POD after surgery, the mean AST/ALT ratio was 1.207; however, analytical findings revealed that no significant differences in 3-year survival rates between the high AST/ALT ratio group and the low AST/ALT ratio group (*P* = 0.978) (Fig. 8).

## Discussion

Our investigation of the association between serum-based hepatic function markers and POD demonstrated that elevated preoperative AST/ALT ratios may serve as an independent risk factor for POD, potentially mediated in part through CSF biomarkers. Furthermore, an AST/ALT ratio exceeding 1.10 was associated with substantially heightened POD risk.

POD, one of the most prevalent postoperative complications in elderly patients, remains incompletely understood pathophysiologically. Multiple clinical factors – including age, infection markers, nutritional status, hepatic/renal function, and CSF biomarkers – have been proposed for POD risk stratification^[20,21]^. While these factors may contribute to POD pathogenesis, their precise mechanistic roles require further experimental and clinical validation. Surgical procedures induce systemic inflammatory responses: tissue trauma triggers peripheral release of proinflammatory cytokines (e.g., IL-6 and TNF-α)^[22]^, which penetrate the BBB to activate central nervous system microglia and astrocytes, initiating neuroinflammation^[23]^. Inflammatory cascades induce neuronal dysfunction and synaptic damage, directly contributing to cognitive decline^[24]^. Increased intraoperative blood loss and prolonged operation time are also risk factors for POD^[25]^, and the intraoperative information in this study was not statistically significant, which may mean that only two surgical methods were counted, and our hospital was specialized in these types of surgery, with less intraoperative blood loss, short operation time, and the role of Enhanced Recovery After Surgery (ERAS). Sum up, there was no significant difference in intraoperative information between the POD and NPOD groups.

Reduced CSF Aβ42 levels may reflect pathological aggregation into amyloid plaques, which activate astrocytes and microglia, perpetuating chronic neuroinflammation^[26]^. Aβ-mediated synaptic dysfunction occurs through membrane binding that disrupts synaptic transmission, exacerbating cognitive impairment. Hyperphosphorylated tau accumulation destabilizes microtubules, leading to cytoskeletal disintegration^[27]^. P-tau competitively inhibits normal tau–microtubule interactions, further impairing neuronal integrity^[28]^. Our previous investigations identified uric acid metabolism as a contributor to neuronal oxidative damage via xanthine oxidase-catalyzed reactive oxygen species generation during purine catabolism^[2]^. This process disrupts the systemic redox balance, potentiating oxidative neuronal injury.

The liver, the primary detoxification organ, facilitates clearance of peripheral metabolites and central nervous system waste products, including proteins implicated in cognitive dysfunction^[15]^. Emerging evidence links hepatic dysfunction to cognitive decline, with liver function indices – such as AST/ALT ratios and ALB – proposed as potential biomarkers^[11]^. Elevated AST/ALT ratios exhibit significant inverse correlations with memory, visuospatial ability, and executive function^[11,29]^. Notably, cognitive impairment cohorts demonstrate markedly reduced ALB and triglyceride levels compared to controls. Wu *et al* reported AST/ALT ratios of 1.17–1.30 in cognitively impaired patients^[11]^, consistent with our findings, suggesting that hepatic injury and inflammatory responses may drive these associations. Higher AST/ALT ratios are associated with lower memory scores and executive function scores. Although the precise mechanism linking AST/ALT to POD remains incompletely understood, emerging evidence suggests potential pathophysiological mechanisms underlying the association between hepatic enzymes and POD. Elevated AST/ALT ratios may also signify heightened oxidative stress, which can impair neuronal function. Hepatic dysfunction may induce metabolic dysregulation, such as hyperammonemia, thereby increasing POD susceptibility. Hepatocytes facilitate the clearance of circulating Aβ through degradation pathways and biliary excretion mechanisms. Age-related hepatocyte depletion reduces hepatic Aβ clearance, potentially exacerbating cerebral Aβ accumulation. Therefore, amyloid and tau pathologies may partially mediate liver–cognition interactions^[12]^. In the ADNI cohort study, 1581 older adults with abnormal liver function showed an elevated AST/ALT ratio, which was associated with increased Aβ deposition in the brain and poor cognition, suggesting that liver clearance may play a role in regulating brain Aβ pathology^[15]^. It has also been demonstrated in animal experiments that a chronic decline in Aβ clearance in the liver increases Aβ deposition in the brain and aggravates tau hyperphosphorylation and neuroinflammation, among others^[12]^. At the same time, it was found that with age, the uptake of Aβ42 in the liver of mice decreased, accompanied by a decrease in lipoprotein receptor-related protein 1 (LRP-1) expression^[12]^. These studies suggest that aging is an important factor leading to the decline of the liver’s ability to clear Aβ, and the decrease in LRP-1 expression may play an important role.

Low-density LRP-1 in the liver is a membrane receptor that mediates the internalization of multiple ligands^[30,31]^. Additionally, LRP-1 regulates several tight junction proteins in BBB endothelial cells^[30]^. Liver sinusoidal endothelial cells (LSECs) are highly specialized scavenger cells; functional LRP-1 is expressed in LSECs and facilitates the clearance of ligands from the bloodstream^[31,32]^. LRP-1, expressed on liver sinusoidal endothelial cells (LSECs), regulates Aβ clearance via blood scavenging^[32]^. In Alzheimer’s disease, diminished LRP-1 expression in hepatic and BBB endothelial cells impairs systemic Aβ elimination, linking hepatic dysfunction to Aβ pathology and cognitive decline.

Elevated AST/ALT ratios correlate with increased cerebral Aβ deposition, elevated CSF P-tau/T-tau levels, and poorer cognition, potentially explaining POD pathogenesis. Elevated AST/ALT ratios are associated with cognitive impairment linked to reduced cerebral glucose metabolism, particularly observed in the bilateral frontal, parietal, and temporal lobes of the brain^[15,33]^. Hypometabolism shifts the tricarboxylic acid cycle toward α-ketoglutarate depletion, favoring glutamate catabolism over synthesis^[34]^. As glutamate serves as the primary neurotransmitter in two-thirds of neocortical and hippocampal synapses, AST/ALT-driven reductions in plasma glutamate may disrupt neurotransmission^[35,36]^. Although preliminary findings mentioned earlier have been reported, the precise signaling pathways and underlying mechanisms remain incompletely elucidated. Further mechanistic investigations utilizing animal models were warranted to delineate these pathways.

The 3-year survival rate was also calculated in this study, and it was found that there was no statistical significance, so the cause of death in patients with POD needs to be further discussed. Individual liver function parameters demonstrated minimal impact on the 3-year survival rate in patients with POD, as survival outcomes were collectively influenced by multifactorial determinants. Future studies will focus on elucidating the interplay of these variables through comprehensive mechanistic investigations. This study originated from PNDABLE, focusing on the predictive effects of lifestyle and biomarkers on POD. Compared with traditional scales, patients have better adherence and objective evaluation.

However, there are several limitations in this study. First, as a single-center investigation with a limited sample size, the findings might not be generalizable to broader populations. Second, while we observed associations between the AST/ALT ratio and CSF-related biomarkers with POD, the analysis precluded definitive conclusions regarding causality or precise biological mechanisms; further experimental studies should be warranted to elucidate these pathways. Finally, the study focused solely on a single liver function parameter (AST/ALT ratio) without a comprehensive evaluation of other Child–Pugh score components (e.g., ALB, bilirubin, and ascites). Priority should be given to recruiting a broader patient population in future studies to improve the reliability and generalizability of the findings. Furthermore, despite the absence of loss to follow-up in patients with POD, however, the limited sample size (especially only five POD patients died in the subgroup analysis) may impair the stability of survival results. Therefore, a larger cohort of patients with POD should be included in follow-up studies with long longitudinal follow-up to verify mortality and assess clinical significance. Finally, beyond aminotransferases, additional liver function parameters should be included in studies to refine POD prediction models and systematically track postoperative recovery trajectories and long-term survival outcomes.

## Conclusion

In conclusion, an elevated AST/ALT ratio in preoperative liver function markers may be a risk factor for POD and may play a mediating role through the CSF biomarkers Aβ42 and Aβ42/P-tau. However, an increase in the AST/ALT ratio did not have a significant effect on the 3-year survival rate of POD patients.

## Data Availability

The data that support the findings of this study are available in the PNDABLE study. According to relevant regulations, the data could not be shared but could be requested from the corresponding author.

## Research registration unique identifying number (UIN)

The PNDABLE study is registered with the China Clinical Trial Registry (registration number: ChiCTR2000033439, registration date: 7 June 2020).
